# Suspected Microvascular Occlusion Syndrome Following Scorpion Envenomation: A Pediatric Case Report

**DOI:** 10.1155/crpe/3601171

**Published:** 2026-11-02

**Authors:** Rana Sadat Saleh, Fateme Jadidi, Reyhane Mohsenifard, Sadra Saleh, Bahare Abtahi

**Affiliations:** ^1^ Department of Pediatrics Isfahan University of Medical Sciences Isfahan Iran mui.ac.ir; ^2^ Skin Diseases and Leishmaniasis Research Center Isfahan University of Medical Sciences Isfahan Iran mui.ac.ir

## Abstract

Scorpion envenomation can cause severe local and systemic complications, including microthrombosis and microvascular occlusion (MVO), which may contribute to tissue ischemia and organ dysfunction. This report describes a pediatric patient who developed progressive edema, ecchymosis, and severe pain after a scorpion sting, despite standard treatment with antivenom, corticosteroids, and analgesics. Persistent symptoms raised concern for possible vascular complications, including localized microthrombosis potentially resulting from venom‐induced endothelial injury, platelet activation, and inflammatory cytokine release. Imaging and clinical findings ruled out compartment syndrome but suggested MVO as a possible mechanism underlying the patient’s worsening condition. This case highlights the importance of early recognition and management of microvascular complications in scorpion envenomation and emphasizes the potential role of anticoagulation and vascular monitoring in selected severe cases.

## 1. Introduction

Scorpions are common arthropods found on every continent except Antarctica. Their stings can cause varying effects, from neuromuscular complications to life‐threatening cardiovascular issues. Annually, around 1.5 million envenomings occur worldwide, resulting in approximately 2600 fatalities, with the elderly, infants, and young children being at higher risk [1, 2]. Scorpion stings are a significant concern in arid regions, including the Middle East, Africa, and Iran. Around 1.2 million people are stung annually, with a higher incidence in warmer months, especially from April to September [3, 4]. Just like many conditions and illnesses, there are some rare complications associated with scorpion stings, such as motor aphasia, stroke, acute kidney injury, pancreatitis, and, in some sporadic cases, microthrombosis [1, 5, 6]. Fatalities may result from the venom’s toxic effects or an anaphylactic reaction [7, 8]. Microvascular thrombosis takes place in the microcirculation due to irregularities in coagulation processes and inflammatory mechanisms, often referred to as thromboinflammation [9]. In this case, we aimed to investigate an uncommon complication of scorpion stings. The patient developed maculopapular lesions on the body, which may indicate microthrombosis in the small vessels secondary to the scorpion sting. This microthrombosis is a type of cutaneous microvascular occlusion syndrome (MVOS).

Cutaneous MVOS is a clinically significant skin lesion characterized by retiform purpura and noninflammatory bland necrosis. It can serve as a marker for identifying associated critical systemic conditions and help prevent potentially life‐ and organ‐threatening complications through timely diagnosis and management [10].

## 2. Case Report

A 12‐year‐old girl was admitted to the hospital with a rare condition. She was brought to the pediatric emergency department with pain, edema, and ecchymosis on her right hand as her chief complaint (Figure 1a), with a sting mark on her fourth finger. She had spent some time sleeping with a blanket that had not been used for 2 months. She was administered with a respiratory rate of 22 breaths per minute, a temperature of 36.8, and a heart rate of 124 bpm.

**FIGURE 1:**
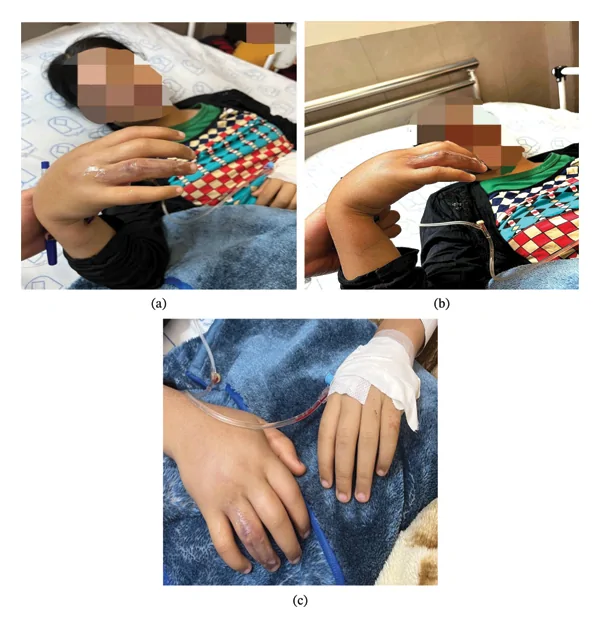
The initial (a), progressive (b), and recovery (c) phases of the injury with nonpitting edema and ecchymosis.

She was admitted to the hospital due to a scorpion sting. Based on endemic data, the shape of the bite mark, and the absence of neurologic symptoms, the sting was suspected to be from the species *Hemiscorpius lepturus* (Gadim). She was given one 5‐mg vial of Razi polyvalent scorpion antivenom, famotidine 40 mg orally every 12 h, hydrocortisone 100‐mg IV every 8 h, hydroxyzine 25 mg orally every 6 h, analgesics, a tetanus vaccine shot, and parenteral antimicrobial treatment including clindamycin (mg/kg/day) and Tazocin (300 mg di piperacillin/kg/day) for a week. No increase in blood pressure or eye nystagmus occurred. Her hand was frequently checked for compartment syndrome and was examined for all five signs (pain, pallor, paresthesia, pulselessness, and paralysis).

After 2 days of admission and despite receiving the proper care, the edema and the ecchymosis spread, and on the patient’s finger was a progressive reticular rash. Although sedatives were prescribed, the severe pain continued, and according to this lesion and the lack of response to the medication, we considered the possibility of compartment syndrome (Figure 1b).

Therefore, an orthopedic consultation and an X‐ray were ordered to rule out compartment syndrome and/or fracture. The results showed a normal compartment pressure of 10 mmHg and no evidence of fracture on X‐ray (Figure 2).

**FIGURE 2:**
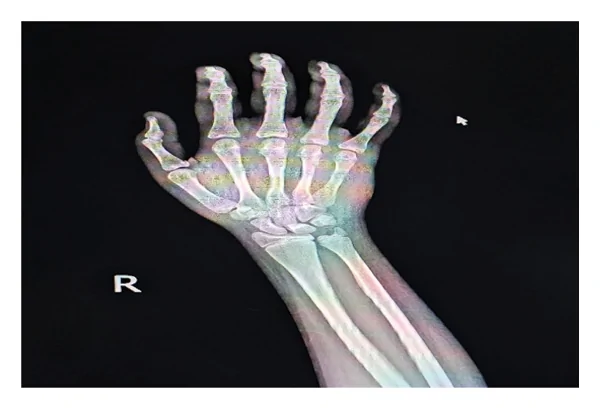
An X‐ray of a right hand related to a scorpion sting, as mentioned in the text above. No sign of fracture was found.

While being treated, there was some recurrence of systemic symptoms such as sinus bradycardia (Figure 3).

**FIGURE 3:**
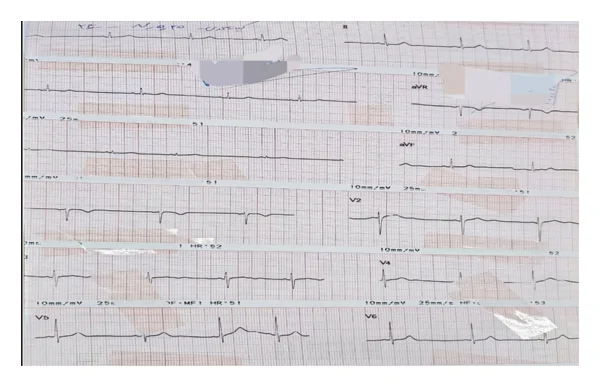
Sinus bradycardia in ECG (rate: 50 beats per minute).

As a result of these findings, electrolytes were checked (Table 1), and pain and fever were controlled. Echocardiography revealed an EF of 70%. No pathological findings were observed in the patient that could indicate the cause of sinus bradycardia; therefore, it is possible that it was due to the systemic effects of the poison. Considering the hand ecchymosis and bradycardia, 2 more of the same 5‐mg vials of Razi polyvalent antivenom were injected into the patient.

**TABLE 1:** Laboratory findings.

Test	Result	Unit	Reference value
White blood cell	12	K/μL	4.5–13.5
Hemoglobin	13.8	g/dL	12–15.5
MCV	88	Femtoliters	80–100
Platelet	356,000	K/μL	214,000–459,000
Red blood cell count	4.23	10^12^/L	3.97–5.01
PT	12	Second	11–13.5
PTT	27	Second	25–35
INR	0.98		0.8–1.2
D‐Dimer	3198	μg/mL	< 500
Fibrinogen	173	mg/dL	200–400
FDP	12	μg/mL	< 5
LDH	968	U/L	140–280
Protein C	95	%	70–130
Protein S	90	%	65–140
Lupus anticoagulant	Neg		
Na	140	mEq/L	135–145
K	4	mEq/L	3.5–5
Mg	2	mEq/L	1.7–2.2
Ca	8.7	mEq/L	8.5–10.5
Phosphate (P)	3.5	mg/dL	2.5–4.5
Albumin	4	g/dL	3.5–5

Although her bradycardia resolved, the edema and ecchymosis persisted. Given the possibility of MVO, her vessels were examined with Doppler ultrasound, focusing on arterial blood flow. The results were normal; therefore, generalized vascular conditions were ruled out. However, considering the lesion’s shape and the persistent reticular rash, nonpitting edema, and ecchymosis, alongside laboratory results such as elevated FDP and D‐dimer, we suggested that it might be a rare complication, such as MVO, rather than a typical case of thrombosis. The persistence of these signs led us to consider vasospasm and related conditions less likely. Based on the antivenom treatment, venom‐induced vasculitis was not expected. Therefore, we started enoxaparin at a therapeutic dose of 2 mg/kg for 15 days, and it proved effective, as the edema and ecchymosis of the hand gradually improved (Figure 1c), which supported our clinical suspicion.

Finally, the patient was discharged from the hospital with a good general condition and stable vital signs, a significant reduction in swelling, redness, and reticular rash of the hand, reduced pain, and a normal HR.

## 3. Discussion

This case report describes a rare complication of scorpion envenomation in a 12‐year‐old girl who presented with pain, edema, ecchymosis, and a reticular rash after a sting to her fourth finger. Despite appropriate initial care, including antivenom administration and symptomatic treatment, the patient’s condition progressed with worsening local symptoms, raising concerns about microvascular complications such as microthrombosis. The clinical course from admission to discharge is summarized in Figure 4.

**FIGURE 4:**
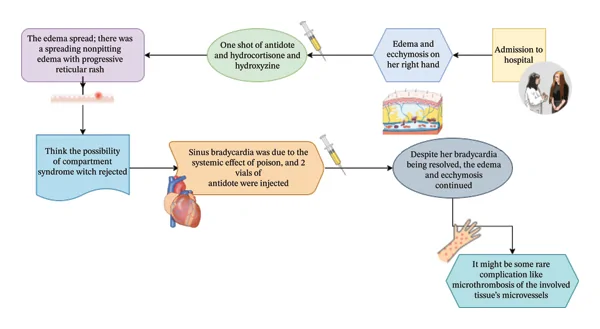
Timeline of symptoms from scorpion sting to microthrombosis.

The mechanism underlying this rare presentation may involve microthrombosis of small vessels. Scorpion venom contains a complex mixture of toxins that can trigger systemic inflammatory responses and coagulation abnormalities. These toxins can induce endothelial dysfunction, platelet aggregation, and thromboinflammatory processes, leading to microcirculatory occlusion. Although Doppler ultrasound showed normal arterial blood flow, it may lack sufficient sensitivity to detect microvascular thrombosis, which may explain the persistence of edema and ecchymosis in this patient.

Based on this patient’s conditions and test results, several differential diagnoses were considered, including compartment syndrome, which was considered unlikely after multiple orthopedic visits, normal results from assessments of the involved tissue’s pressure, and the absence of signs such as pain, paresthesia, pallor, paralysis, and pulselessness; vasospasm, which was deemed less likely given the persistence of signs such as the reticular rash and nonpitting edema; venom‐induced complications, which were thought to be unlikely given the antivenom treatment prescribed for the patient with an appropriate dose and type; fracture, which was ruled out using an X‐ray image acquired from the site of the lesion; microthrombosis, which was ruled out based on a normal Doppler test result; and MVO, which could be the most likely based on the presence and persistence of the reticular rash, the nonpitting edema, laboratory results such as elevated D‐dimer and FDP and decreased fibrinogen, and the significant response to the treatment.

Management of this patient included multiple interventions to address both systemic and localized effects. In addition to the initial antivenom and symptomatic treatments, such as analgesics and antibiotics, anticoagulation therapy was initiated to target suspected microthrombosis. The anticoagulant regimen proved effective, as the patient’s symptoms improved significantly, and she was discharged with stable vitals and reduced swelling and pain.

The novelty of this case lies in the diagnostic challenge posed by uncommon conditions, unlike in other scorpion stings where symptoms often include neuromuscular or cardiovascular manifestations, such as arrhythmias and myocarditis. In most cases, Doppler imaging and routine coagulation tests are sufficient to rule out vascular complications, and severe symptoms are often systemic rather than localized. However, in cases such as this one, where the complication is rare, diagnosis relies primarily on clinical suspicion and assessment rather than on imaging or Doppler. There are other complications requiring the same approach, such as lateral medullary syndrome and acute coronary syndrome, that have also been reported in the literature.

Scorpion envenomation is associated with several problems, one of which is heart complications, including cardiac arrhythmias and myocarditis, which is one of the causes related to scorpion envenomation death [11]. It has also been shown in a study that there is a significant relationship between scorpion stings and lateral medullary syndrome [12]. The relationship between scorpion envenomation and acute coronary syndrome is mentioned in a few articles, and most of the patients with a scorpion sting had a normal echocardiogram [13]. Although cases of envenomation‐induced peripheral vascular complications are rare in scientific literature, there are reports of these complications associated with venomous bites and stings from snakes, spiders, and scorpions, especially in pediatrics, including intravascular coagulopathy, local swelling, edema, hematoma, and compartment syndrome [14, 15].

## 4. Limitations

A significant limitation of this report is the inability to confirm MVO with Doppler imaging and the lack of a dedicated imaging method or module to detect this complication. Additionally, this is a single case, particularly in this endemic area, which limits the generalizability of the findings. Another major limitation is that, although we ruled out other differential diagnoses to the best of our ability, we ultimately had to rely primarily on clinical suspicion and, later, on a positive response to the prescribed treatment rather than on paraclinical findings and criteria, since there are no guidelines for such cases. Another limitation we would like to address is that, due to a lack of definitive evidence, such as a picture or the scorpion itself, we were not able to definitively identify the species of the scorpion and could only suspect the species based on the bite mark and the symptoms the patient presented with. Further studies involving larger cohorts and advanced diagnostic modalities are needed to validate the association between scorpion stings and microvascular thrombosis.

## 5. Conclusion

The significant positive response to the prescribed treatment shows that despite the diagnostic challenges in this case, nonpitting edema and a reticular rash following a scorpion sting, along with elevated D‐dimer and FDP levels, can serve as important clinical indicators for pediatricians and emergency medical personnel, prompting consideration of rare complications such as MVO. Early recognition and timely anticoagulant therapy may prevent further symptom progression and improve outcomes in similar cases; medical team alertness and suspicion toward such complications, along with strict follow‐up on treatment response, are deemed crucial for effective diagnosis and treatment.

## Funding

No funding was received for this manuscript.

## Conflicts of Interest

The authors declare no conflicts of interest.

## Data Availability

The data that support the findings of this study are available on request from the corresponding author. The data are not publicly available due to privacy or ethical restrictions.
